# miR-221/222 induces pancreatic cancer progression through the regulation of matrix metalloproteinases

**DOI:** 10.18632/oncotarget.3686

**Published:** 2015-03-29

**Authors:** Qinhong Xu, Pei Li, Xin Chen, Liang Zong, Zhengdong Jiang, Ligang Nan, Jianjun Lei, Wanxing Duan, Dong Zhang, Xuqi Li, Huanchen Sha, Zheng Wu, Qingyong Ma, Zheng Wang

**Affiliations:** ^1^ Department of Hepatobiliary Surgery, First Affiliated Hospital Medical College of Xi'an Jiaotong University, Xi'an, Shaanxi, China; ^2^ Department of Thoracic Surgery, Tangdu Hospital, Fourth Military Medical University, Xi'an, Shaanxi, China; ^3^ Department of General Surgery, First Affiliated Hospital Medical College of Xi'an Jiaotong University, Xi'an, Shaanxi, China

**Keywords:** miR-221/222, pancreatic cancer, Matrix metalloproteinases

## Abstract

MicroRNAs are involved in the initiation and progression of pancreatic cancer. In this study, we showed that miR-221/222 is overexpressed in pancreatic cancer. MiR-221/222 overexpression significantly promoted pancreatic cancer cell proliferation and invasion while inhibiting apoptosis. The expression of the matrix metalloproteinases (MMPs) MMP-2 and MMP-9 was increased in miR-221/222 mimic-transfected pancreatic cancer cells. Validation experiments identified TIMP-2 as a direct target of miR-221/222. These data indicate that overexpressed miR-221/222 may play an oncogenic role in pancreatic cancer by inducing the expression of MMP-2 and MMP-9, thus leading to cancer cell invasion.

## INTRODUCTION

Difficult early diagnosis, local aggression and rapid progression are the features of pancreatic cancer, which has the poorest overall survival rate among all human cancers [1]. The overall median survival for patients with pancreatic cancer is 2–8 months, and the 5-year survival rate is less than 5% [2]. The extremely high mortality approaches 100% due to difficulties in early clinical diagnosis and the lack of effective treatment methods [3]. Only approximately 10% to 20% of patients with pancreatic cancer are suitable for surgery at the time of diagnosis because of tumor invasion and metastasis [4]. Therefore, understanding the molecular mechanisms of pancreatic cancer that are associated with its aggressiveness and high propensity for metastasis and developing novel therapeutic targets for pancreatic cancer are imperative.

MicroRNAs are a type of small non-coding RNA that regulate gene expression by affecting mRNA stability and protein translation [5, 6]. A previous study reported that approximately 60% of protein-encoding genes could be regulated by miRNAs [7]. The deregulation of miRNAs play important roles in cancer occurrence and development [8]. miRNAs are abnormally expressed in several tumors and can play a role as an oncogene or tumor suppressor gene [9]. miR-203 inhibits glioma cell migration by disrupting the Robo1/ERK/matrix metalloproteinase (MMP)-9 signaling axis [10], and MMP-2 is a miR-29b target in prostate cancer cells [11]. Some studies have shown the deregulation of miRNAs in pancreatic cancer tissues using miRNA array technology and real-time PCR analysis [12-14]. miR-221 and miR-222 are expressed from a single transcript that is found on the X chromosome. Both miRNAs possess the same sequence, which is an evolutionarily conserved, short region at the 5′ end through which they bind target sites in the mRNA's 3′ UTR [15, 16]. As oncogenes, miR-221/222 has been studied in many epithelial cancers, including glioma, prostate carcinoma, hepatocellular cancer, lung cancer and breast cancer [1, 17-24]. The expression of miR-221 is significantly up-regulated in pancreatic cancer cell lines and tumor tissues compared with normal pancreatic duct epithelial cells and normal pancreatic tissues [25].

MMPs are closely related to cell migration and invasion and are regarded as a marker of malignant tumor invasion and metastasis [26, 27]. MMPs may participate in the initial steps to degrade the extracellular matrix (ECM) surrounding endothelial cells [28]. Among the MMPs, MMP-2 and MMP-9 have been implicated in human cancer invasion [29], and we investigated the relationship between these two genes and miR-221/222 in this study.

In this study, we investigated the expression of miR-221/222 in pancreatic cancers. In addition, we confirmed whether miR-221/222 acts as an oncogene in pancreatic tumorigenesis and clarified its possible mechanism.

## RESULTS

### miR-221/222 were upregulated in PDAC tissue and cell lines

**Figure 1:**
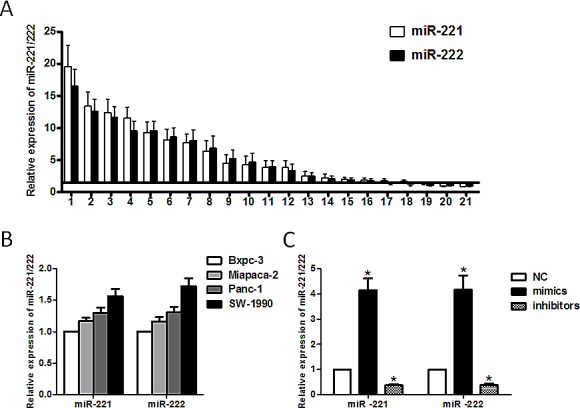
Expression of miR-221/222 in pancreatic cancer tissues and cell lines (**A**) miR-221/222 were detected in 21 pancreatic cancer patients by qRT-PCR. The data are presented for those genes with a >1.5-fold increase in pancreatic cancer tissues relative to non-tumor adjacent tissues. (**B**) The relative expression of miR-221/222 in pancreatic cancer cell lines (Bxpc-3, Miapaca-2, Panc-1 and SW-1990) was detected by qRT-PCR. (**C**) The miR-221/222 mimics or inhibitors transfection efficiency was confirmed by qRT-PCR. The results showed that the levels of miR-221/222 in transformed Panc-1 cells increased by 4.15 ± 0.46/4.16 ± 0.57-fold or were reduced by 0.38 ± 0.05/0.39 ± 0.06-fold following transfection with miR-221/222 mimics or miR-221/222 inhibitors, respectively. U6 shRNA was used as an internal control. Data are expressed as the mean ± SD for three separate experiments performed in duplicate (*p < 0.05).

The expression level of miR-221/222 was significantly higher in PDAC tissues than in paired adjacent normal pancreatic tissues (Figure 1A). Among 21 patients, we found that 18 of 21 cases had a more than 1.5-fold increase in miR-221 in the pancreatic cancer tissue compared with their paired adjacent pancreatic tissue. Similar results were observed in the miR-222 group. In cultured cell lines, the expression of miR-221/222 was much higher in invasive pancreatic cancer cell lines (SW-1990, Panc-1 and Miapaca-2) than in a nonaggressive cell line (Bxpc-3) (Figure 1B). These findings indicated that miR-221/222 might play a role in pancreatic cancer invasion. Among the cell lines, we selected Panc-1 and SW-1990 cells, which have the high expression levels of miR-221/222, to evaluate the function of these miRNAs.

To observe the function of these miRNAs, we applied miRNA mimics and inhibitors. Upon transfection of miR-221/222 mimics, the expression level of miR-221/222 increased more than 4-fold compared with transfection of a negative control. In contrast, the expression miR-221/222 decreased up to 40% when miRNA inhibitors were transfected (Figure 1C).

### miR-221/222 can alter pancreatic cancer cell proliferation

**Figure 2:**
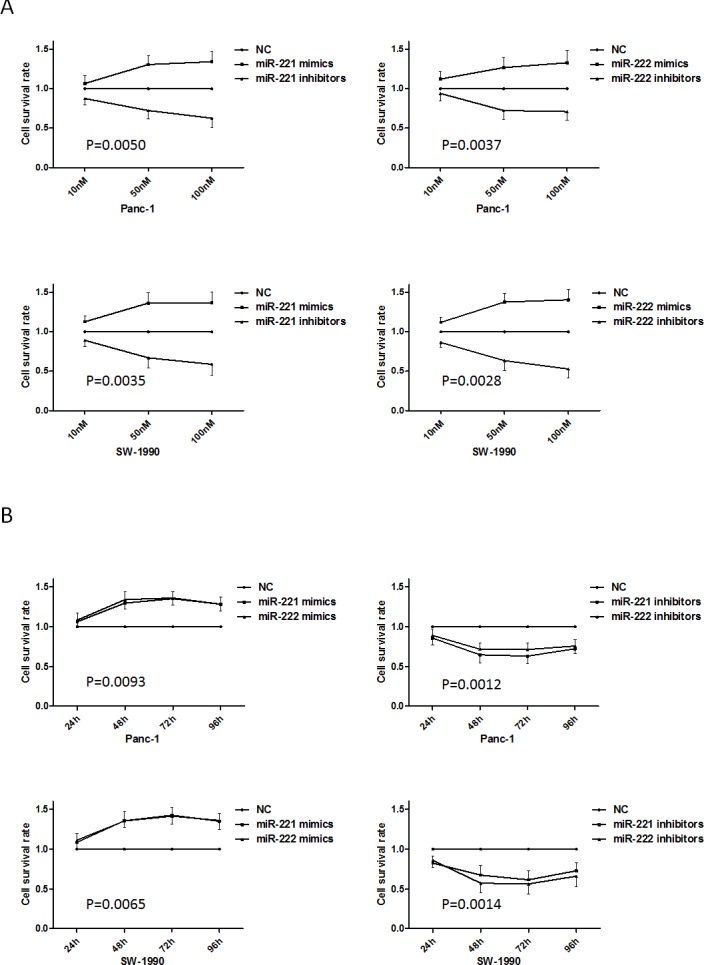
Up-regulated or down-regulated miR-221/222 levels in pancreatic cancer cell lines induce growth promotion and inhibition (**A**) The MTT assay showed growth promotion or inhibition in Panc-1 and SW-1990 cells transfected with various concentrations of miR-221/222 mimics or inhibitors. At 48 h after transfection, the growth inhibition in Panc-1 cells transfected with miR-221/222 inhibitors was 64.65 ± 10.47% and 71.73 ± 8.10%. Similarly, SW-1990 cells showed 67.25 ± 12.23% and 57.20 ± 12.45% decreases by reducing miR-221/222 levels. Growth promotion in Panc-1 cells transfected with miR-221/222 mimics was 129.68 ± 7.24% and 134.06 ± 10.33%. Similarly, SW-1990 cells showed 135.63 ± 8.50% and 135.35 ± 12.13% increases in growth by enhancing miR-221/222 levels. (**B**) The MTT assay showed growth promotion or inhibition in Panc-1 and SW-1990 cells transfected with miR-221/222 mimics or inhibitors at different time points.

To investigate the influence of miR-221/222 on cell proliferation, the MTT assay was used. MTT assays (both dose-dependent and time-dependent) illustrated significant increases or decreases in Panc-1 and SW-1990 cells after transfection of miR-221/222 mimics or miR-221/222 inhibitors, respectively (Figure 2). At 48 h after transfection, Panc-1 and SW-1990 cells were significantly inhibited when transfected with miR-221/222, and the maximum growth inhibition appeared at 48 h after transfection (*p* < 0.05) compared with the miRNA mimics.

### miR-221/222 can modulate the cell cycle *in vitro*

**Figure 3:**
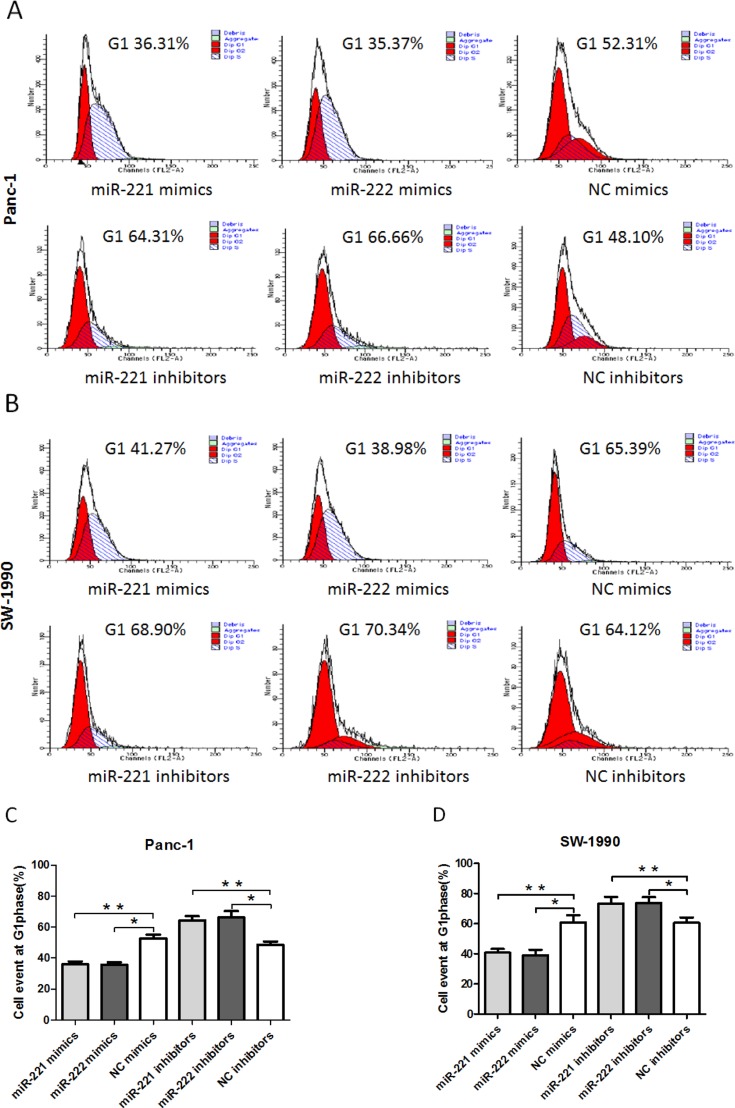
A FACS cell cycle analysis showed a G1 accumulation in Panc-1 and SW-1990 cells at 48 h after transfection of miR-221/222 mimics (**A** and **B**) Cell cycle analysis was performed by flow cytometry in Panc-1 or SW-1990 cells at 48 h after transfection of miR-221/222 mimics, inhibitors or negative controls. miR-221/222 mimic-transfected cells showed a decrease in the number of G1 phase cells (35.97 ± 1.93%/35.62 ± 1.73% in Panc-1 cells and 40.83 ± 2.43%/39.08 ± 3.65% in SW-1990 cells). In contrast, the miR-221/222 inhibitor-transfected cells had a significantly higher proportion of cells in G1 phase (64.19 ± 2.86%/66.16 ± 4.16% in Panc-1 cells and 73.42 ± 4.37%/73.81 ± 3.86% in SW-1990 cells). (**C** and **D**) Graphical representation of the FCM analysis in A and B (*p < 0.05). The data are expressed as the mean ± SD of three independent experiments.

To further explore the potential effect on cell proliferation in Panc-1 and SW-1990 cells, we used flow cytometry to analyze the cell cycle (Figure 3). After 48 h, the cells were harvested. miR-221/222 mimic-transfected cells (Panc-1 and SW-1990) showed a decrease in the number of G1 phase cells. In contrast, the miR-221/222 inhibitor-transfected cells had a significantly higher proportion of cells in the G1 phase compared with the negative control group (*p* < 0.05).

### miR-221/222 can induce anti-apoptosis

**Figure 4:**
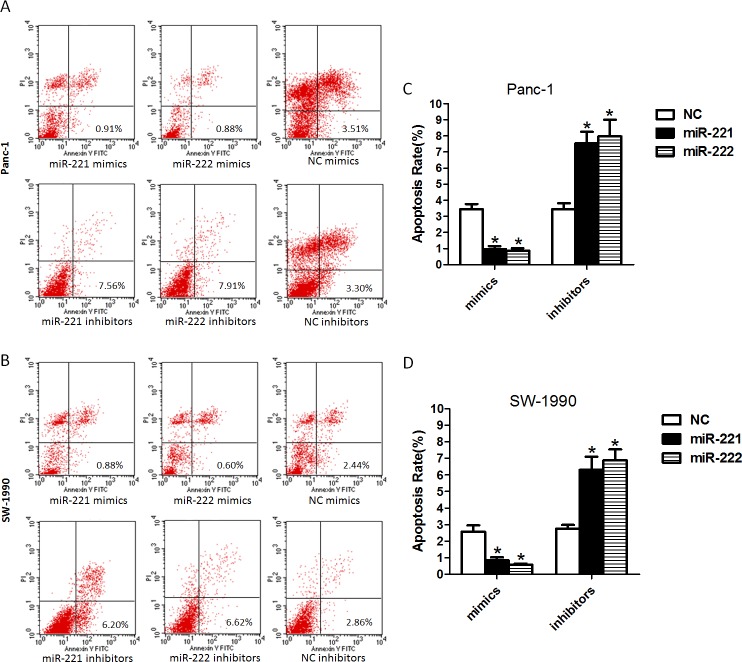
Up-regulated or down-regulated miR-221/222 levels in pancreatic cancer cell lines decrease or increase the rate of apoptosis (**A** and **B**) Cell apoptosis was analyzed by FCM in Panc-1 or SW-1990 cells at 48 h after transfection of miR-221/222 mimics, inhibitors or negative controls. The data showed that a significantly lower apoptotic rate was found in the miR-221/222 mimic-transfected Panc-1 cells compared with their controls (0.97 ± 0.32%/0.88 ± 0.26%). A similar phenomenon was observed in the SW-1990 cells (0.84 ± 0.31%/0.57 ± 0.14%). The apoptotic rates of the miR-221/222 inhibitor-transfected cells were 7.55 ± 1.23%/7.97 ± 1.81% in Panc-1 cells and 6.31 ± 1.36%/6.88 ± 1.16% in SW-1990 cells. (**C** and **D**) Ratio of apoptosis in cells transfected with oligoribonucleotides (*p < 0.05). The data are expressed as the mean ± SD of three independent experiments.

To investigate the effect of miR-221/222 mimics or miR-221/222 inhibitors on apoptosis, PI and Annexin V double staining assays were performed (Figure 4). At 48 h post-transfection, a significantly lower apoptotic rate was observed in the miR-221/222 mimic-transfected Panc-1 cells compared with the controls. A similar phenomenon was found in SW-1990 cells. In contrast, the apoptotic rates of the miR-221/222 inhibitor-transfected Panc-1 and SW-1990 cells were significantly increased compared with those in the negative control group. These results suggest that miR-221/222 inhibitors could inhibit cell growth and increase the induction of apoptosis. In addition, miR-221/222 mimics promote cell growth and decreased apoptosis.

### miR-221/222 modulates cell invasion and promotes relative gene expression

**Figure 5:**
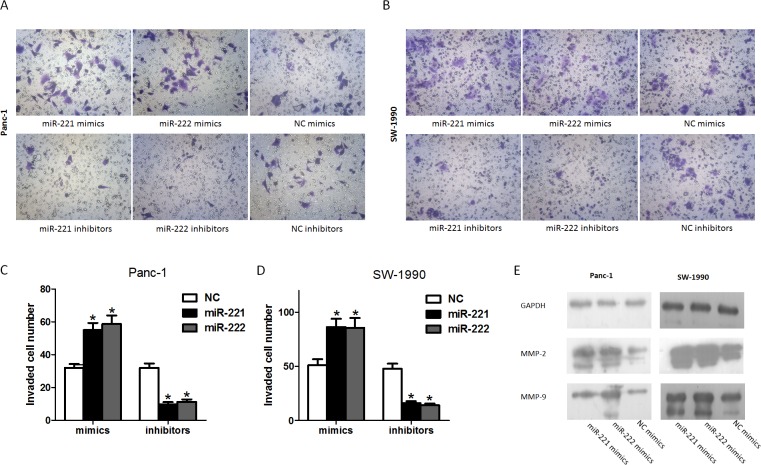
miR-221/222 promotes the invasion of pancreatic cancer cells (**A**) Invasion after transfection of Panc-1 cells with miR-221/222 mimics or inhibitors and their negative controls. (**B**) Invasion after transfection of SW-1990 cells with miR-221/222 mimics or inhibitors and their negative controls. (**C** and **D**) Graphical representation of the transwell assays in A and B (*p < 0.05). The data are expressed as the mean ± SD of three independent experiments. (**E**) Cells were transfected with miR-221/222 mimics or negative controls. MMP2 and MMP9 protein levels were detected by western blot assays. GAPDH protein was assayed as a control.

To investigate whether miR-221/222 is associated with cell invasion, we transfected Panc-1 and SW-1990 cells with miR-221/222 mimics or inhibitors. Based on the Matrigel invasion assay shown (Figure 5A-5D), the invasion ability of miR-221/222 mimic-transfected cells was significantly increased compared with the negative controls. Moreover, silencing miR-221/222 significantly decreased cell invasion compared with cells treated with negative control oligonucleotides.

To detect the relative expression of invasion-related factors, we selected two known genes, MMP-2 and MMP-9, and detected their expression in cells transfected with miR-221/222 mimics and their negative controls. In cells transfected with miR-221/222, the data showed that MMP-2 and MMP-9 were up-regulated compared with cells transfected with NC (Figure 5E). We conclude that miR-221/222 enhances MMP-2 and MMP-9 expression in Panc-1 and SW-1990 cells, which could influence the cell's behavior.

### TIMP-2 may be a downstream target gene of miR-221/222

**Figure 6:**
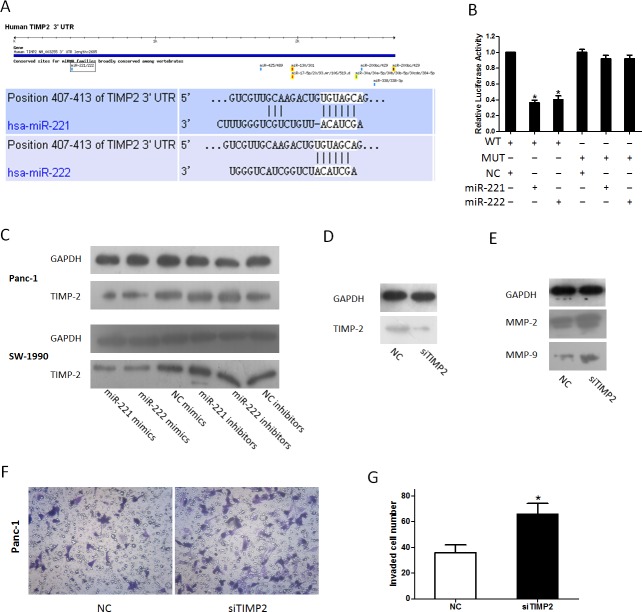
miR-221/222 targets TIMP-2 (**A**) Software prediction of miR-221/222 determines a potential binding site in the TIMP-2 3′ UTR. The nucleotide sequence illustrates the predicted base pairing between miR-221/222 and the TIMP-2 3′ UTR. (**B**) *In vitro* luciferase reporter assay in HEK293 cells. miR-221/222 mimics or inhibitors and their negative controls were co-transfected with reporter plasmids containing the wild-type (wt) TIMP-2 3′UTR and mutant (mu) TIMP-2 3′UTR (*p < 0.05). (**C**) Cells were transfected with miR-221/222 mimics or inhibitors, and TIMP-2 protein levels were detected by western blot assays. GAPDH protein was assayed as a control. (**D**) siTIMP-2 could inhibit the expression of TIMP-2. (**E**) Panc-1 cells were transfected with siTIMP2; protein levels of MMP-2 and MMP-9 were detected by western blot assays. (**F** and **G**) Inhibition of TIMP-2 by siTIMP2 mimics miR-221/222-induced invasion. (*p < 0.05).

In our previous study on miRNAs [30], we found that TIMP-2 is the target of miR-106a. Combined with the results of a miRNA target prediction program (TargetScan) (Figure 6A), we hypothesized that miR-221/222 could also target TIMP-2 mRNA, which inhibits MMPs. To verify our predicted target of miR-221/222, HEK293 cells were co-transfected with a reporter plasmid (WT-TIMP-2 3′ UTR or its mutant form MUT-TIMP-2 3′ UTR) and miR-221/222 mimics or a negative control as we previously reported [30]. A Dual-Luciferase Reporter Assay showed that the luciferase activity in cells co-transfected with WT-TIMP-2 3′ UTR and miR-221/222 mimics was reduced by nearly 36.33 ± 3.06% (miR-221)/40.33 ± 4.73% (miR-222) (Figure 6B). A western blot analysis was performed to detect changes in miR-221/222-mediated repression of endogenous TIMP-2 in Panc-1 and SW-1990 cells. At 48 h after transfection, miR-221/222 overexpression resulted in a significant decrease in endogenous TIMP-2 protein levels, whereas TIMP-2 expression was elevated in cells with reduced levels of miR-221/222 (Figure 6C). To further investigate the role of TIMP-2 in modulating invasion, we explored the expression of MMP-2 and MMP-9 after transfection with siTIMP-2. The data showed that the expression of MMP-2 and MMP-9 was significantly up-regulated after transfection with siTIMP-2, and the results correlated with the miR-221/222 mimic group (Figure 6D and 6E). In addition, a transwell assay showed a similar result (Figure 6F and 6G).

## DISCUSSION

Pancreatic cancer is the fourth leading cause of cancer death in the United States. Survival is very low because pancreatic cancer is locally aggressive and progresses rapidly [1]. In addition, 80–85% of patients present with advanced unresectable disease. Many studies have found that various factors are involved in the invasiveness of pancreatic cancer. miRNAs have been extensively studied in recent years. Small non-coding RNA molecules play an important role in tumorigenesis and development. A single miRNA can influence the expression of several thousand genes, thus controlling one-third of the human genome [31]. In addition, abundant studies on miRNAs have described that miRNAs can function as oncogenes or tumor suppressor genes and closely relate to the development and progression of different tumors [32]. Recent studies confirmed that miR-494 inhibits PDAC cell growth, migration, and metastasis by negatively regulating FOXM1 expression and subsequently attenuating the β-catenin signaling pathway [33]. The down-regulation of miR-221 inhibits the proliferation of pancreatic cancer cells through the up-regulation of PTEN, p27kip1, p57kip2, PUMA and Ki67. miR-221 could be an oncogenic miRNA and a prognostic factor for the poor survival of patients [34-36]. A specific miRNA can regulate the expression of genes involved in multiple cellular pathways, serving as a potent therapeutic target. Thus, the complete understanding of the role of a specific miRNA is very important for elucidating the mechanism of tumor development.

In this study, we evaluated the carcinogenic role of miR-221/222 in pancreatic cancer. The miRNAs analyzed in this study were selected from a comparison of miRNA expression patterns in pancreatic cancer tissues and adjacent benign tissues [37]. Our findings showed that miR-221/222 was overexpressed in pancreatic cancer, as determined by qRT-PCR. Moreover, the overexpression of miR-221/222 was also found in other tumors in a previous report [23]. A close relationship was found between miR-221/222 expression and pancreatic cancer cell lines. A previous study reported that miR-221/222 was upregulated in Miapaca-2 compared with normal human pancreatic ductal epithelium [14]. Our results showed that miR-221/222 expression was increased with enhanced metastatic propensity in pancreatic cancer cell lines. These data suggest that miR-221/222 overexpression is common in human pancreatic cancer and may have a strong association with carcinogenesis and invasion.

Considering the important role of miR-221/222 in pancreatic cancer, we observed the cell behavior of pancreatic cancer cells. The MTT assay showed that cell viability was significantly inhibited by silencing endogenous miR-221/222, but the recovery of the miR-221/222 levels could relieve this phenomenon and further stimulate tumor cell growth. To further explore the potential mechanism underlying the decreased cell viability of Panc-1 or SW-1990 cells, the cell cycle and cell apoptosis were analyzed by flow cytometry (FCM). miR-221/222 inhibitor-transfected cells showed an increase in the number of G1 phase cells compared with NC-transfected cells. The result indicated a reduction in miR-221/222-induced cell cycle arrest at the G1 phase in Panc-1 and SW-1990 cells. The results of the flow cytometry assay also indicated that apoptosis was promoted by blocking endogenous miR-221/222 in both Panc-1 and SW-1990 cells. Additionally, the upregulation of miR-221/222 blocked apoptosis and promoted cell survival. Thus, miR-221/222 could decrease cell cycle arrest at the G1 phase and cellular apoptosis and then promote cell proliferation in pancreatic cancer. In the transwell assay, the results showed that miR-221/222 could positively influence the invasiveness of pancreatic cancer cells. In addition, we detected that the expression of MMP-2 and MMP-9 was upregulated after miR-221/222 transfection. Because MMP-2 and MMP-9 are important factors that influence cell invasion, we showed that pancreatic cancer cell invasion is dependent on miR-221/222 regulation.

In a previous study [30], we found that miR-106a could promote pancreatic cancer cell invasion by upregulating MMP-2 and MMP-9, and we verified that TIMP-2 was the target of the miRNA. Interestingly, we also found that TIMP-2 may be the target of miR-221/222 through target gene prediction software. To confirm the target gene, we used a dual luciferase 3′ UTR reporter assay and western blot assay. TIMPs are a family of proteins that naturally inhibit the activity of MMPs [38]. The activity of MMPs is reversibly inhibited by TIMPs at a 1:1 stoichiometric ratio in tissues. The impact of TIMPs is essential for the homeostasis of the ECM. An imbalance between MMPs and TIMPs is pivotal for many physiological processes in tissues, including the tumor microenvironment [39]. In pancreatic cancer, MMPs are considered invasive markers and could be attractive targets for anticancer therapy [40-42]. TIMP-2, a member of the TIMP gene family, not only has an inhibitory role against metalloproteinases but also directly suppresses the proliferation of endothelial cells. As a natural target of TIMPs, MMP-2 and MMP-9 play important roles in tumor invasiveness [43]. Using a dual luciferase 3′ UTR reporter assay, we verified that miR-221/222 facilitates pancreatic cancer invasion by directly targeting TIMP-2, which is an inhibitor of MMPs. The data show that highly expressed miR-221/222 could inhibit TIMP-2 and further upregulate the expression of MMP-2 and MMP-9, which would promote the invasion of pancreatic cancer cells. In contrast, we found the down-regulation of TIMP-2 and inhibition of invasion after silencing the endogenous miR-221/222. Thus, TIMP-2 is a pivotal target of miR-221/222-induced pancreatic cancer cell invasion.

In summary, we confirmed the expression of miR-221/222 in primary human pancreatic tissues and their oncogenic role in pancreatic cancer cells. Our data showed that miR-221/222 expression was upregulated in human pancreatic cancer, which inhibited apoptosis and promoted proliferation and invasion in pancreatic cancer cells. Furthermore, we observed an increase in MMP-2 and MMP-9 after mimic transfection and showed that TIMP-2 is a direct functional target of miR-221/222. miR-221/222 promoted cell proliferation by decreasing G1 phase cells and decreasing apoptotic cells. Moreover, miR-221/222 directly bound TIMP-2, upregulated MMP-2 and MMP-9 expression and promoted cell invasion. In conclusion, miR-221/222 could be a promising target for the treatment of pancreatic cancer.

## MATERIALS AND METHODS

### Specimens and cell culture

Twenty-one pancreatic tissues and matched adjacent tissue were obtained from patients who were undergoing radical surgical resection at the First Affiliated Hospital of Xi'an Jiaotong University from 2010 to 2012. The histopathological type of pancreatic cancer was confirmed as pancreatic ductal adenocarcinoma. These patients did not undergo local or systemic adjuvant therapy before operation. The tissue samples were immediately snap-frozen and stored at −80°C. The study was approved by the ethics committee and the human research review committee of Xi'an Jiaotong University, and each subject provided a signed informed consent before participating in this study.

The human pancreatic cancer cell lines Panc-1, Miapaca-2, Bxpc-3, and SW-1990 and the human embryonic kidney cell line (HEK) 293 were purchased from the American Type Culture Collection (Manassas, VA). The Panc-1, Miapaca-2 and HEK 293 cell lines were cultured in Dulbecco's modified Eagle's medium (DMEM) (HyClone), Bxpc-3 cells were cultured in RPMI-1640 medium, and the SW-1990 cells were cultured in L15 medium. All media were supplemented with 10% fetal bovine serum (FBS) and 1% (wt/vol) antibiotic (penicillin/streptomycin). The cells were cultured at 37°C in a 5% CO_2_ atmosphere in a humidified incubator.

### Oligonucleotide synthesis and transfection

miR-221/222 mimics, miR-221/222 inhibitors, siRNA-TIMP2 (siTIMP-2) and negative control oligonucleotides were purchased from GenePharma (Shanghai, China). The sequences are as follows:

miR-221 mimic, 5′-AGCUACAUUGUCUGCUGGGUUUC-3′; miR-222 mimic, 5′-AGCUACAUCUGGCUACUGGGU-3′; mimic negative control, 5′-UUCUCCGAACGUGUCACGUTT-3′; miR-221 inhibitor, 5′-GAAACCCAGCAGACAAUGUAGCU-3′; miR-222 inhibitor, 5′-ACCCAGUAGCCAGAUGUAGCU-3′; inhibitor negative control, 5′-UUCUCCGAACGUGUCACGUTT-3′; and siTIMP-2, 5′-GCAAUGCAGAUGUAGUGAUTT-3′.

Lipofectamine 2000 (Invitrogen) was used for transfection according to the manufacturer's instructions.

### Quantitative real-time PCR analysis

Total RNA was extracted from specimens and cultured cells following the protocol of TRIzol reagent (Invitrogen). A spectrophotometer (NanoDrop Technologies) was used to determine the quality and concentration of RNA by measuring the absorbance at 260 nm (A_260_) and 280 nm (A_280_). We used the PrimeScript® RT Reagent Kit Perfect Real Time (TaKaRa) to reverse transcribe total RNA into cDNA. For miRNA, reverse transcription was completed by miRNA-specific stem-loop primers. The stem-loop primer set for miRNA reverse transcription is described in Table 1. Briefly, 500 ng (1 μl) of total RNA was mixed with 2 μl of 5× PrimeScript® Buffer, 0.5 μl of PrimeScript® RT Enzyme Mix I, 0.5 μl of miRNA-specific stem-loop primer and 6 μl of RNase Free dH_2_O. Then, the mixture was incubated at 37°C for 15 min and 85°C for 5 sec before being maintained at 4°C.

**Table 1:** Primers for the detection of miR-221/222 by qRT-PCR

miR-221	SL: 5′-GTCGTATCCAGTGCGTGTCGTGGAGTCGGCAATTGCACTGGATACGACGAAACCC-3′
	F: 5′-ATCCAGTGCGTGTCGTG-3′
	R: 5′-TGCTAGCTACATTGTCTGCT-3′
miR-222	SL: 5′-GTCGTATCCAGTGCGTGTCGTGGAGTCGGCAATTGCACTGGATACGACACCCAGT-3′
	F: 5′- ATCCAGTGCGTGTCGTG-3′
	R: 5′- TGCTAGCTACATCTGGCT-3′
U6	F: 5′-CTCGCTTCGGCAGCACA-3′
	R: 5′-AACGCTTCACGAATTTGCGT-3′

The levels of the miRNAs were measured with a SYBR Green Real-time PCR Master Mix (TaKaRa). The primer set for qRT-PCR is listed in Table 1.

Real-time PCR was performed using an iQ5 Multicolor Real-Time PCR Detection System (Bio-Rad, Hercules, CA), and fold changes in gene expression were calculated using the 2^−^-ΔΔ^Ct^ method. The PCR conditions were as follows: 30 s at 95°C followed by 40 cycles of 95°C for 5 s and 60°C for 30 s.

### MTT assay

The MTT assay was used to measure cell viability after transfection in either a dose-dependent manner or time-dependent manner. In the dose-dependent proliferation assay, cells were seeded into 96-well culture plates. Then, either miR-221/222 mimics (10 nM, 50 nM and 100 nM) or miR-221/222 inhibitors (10 nM, 50 nM and 100 nM) with the corresponding control were transfected after 24 h. Forty-eight hours after transfection, MTT (5 mg/ml, 20 μl/well) was added, and the culture plate was incubated for 4 h at 37°C. Then, the supernatant was removed, and 150 μl/well dimethyl sulfoxide (DMSO) was added. The optical density (OD) was measured at a wavelength of 490 nm. In the time-dependent proliferation assay, cells were transfected with either miR-221/222 mimics (50 nM) or miR-221/222 inhibitors (100 nM). Then, the cells were cultured for 24 h, 48 h, 72 h and 96 h. Viable cell numbers were measured by the MTT assay. The data were derived from triplicate samples of at least three independent experiments.

### Cell cycle analysis

FCM (Becton Dickinson, USA) was used to perform the cell cycle analysis. Seventy-five percent ethanol was used to fix the transfected and control cells. The fixed cells were treated with 25 μg/ml DNase-free RNase A and stained with 50 μg/ml propidium iodide (PI; Sigma, USA).

### FCM for apoptosis assay

After 48 h, the cells from each well were harvested, stained using the Annexin V-FITC/PI Kit (BD Biosciences) and analyzed with an EPICS ALTRA flow cytometer (Beckman Coulter). The tests were repeated in triplicate.

### Matrigel invasion assay

The cells were transfected for 48 h and then treated with serum-free medium that was placed in the upper chamber of a filter (pore size, 8.0 μm; Millipore, Billerica, USA) coated with Matrigel (BD Biosciences, Franklin Lakes, USA). Then, 500 μl of DMEM containing 15% FBS was added into the lower chamber. The cells were incubated for 24 h at 37°C. After 24 h of incubation, a cotton swab was used to scrape the cells remaining in the upper chamber. Then, the filter was fixed in methanol and stained with crystal violet. Invasion ability was evaluated by counting the migrated cells in five randomly selected fields under a light microscope.

### Western blotting analysis

The cells were lysed by lysis buffer on ice with protease inhibitors (Roche, Penzberg, Germany). The proteins were separated on 10% SDS-PAGE gels and transferred to PVDF membranes (Millipore). Then, the membranes were blocked with 5% skim milk and incubated with rabbit anti-human antibodies against TIMP-2, MMP-2, or MMP-9 (Santa Cruz Biotechnology) or GAPDH (Cell Signaling) overnight at 4°C. The membranes were hybridized with a goat anti-rabbit secondary antibody for 1 h at room temperature. Specific binding was detected using a chemiluminescence system (Millipore).

### Dual luciferase 3′ UTR reporter assay

To investigate miR-221/222 target genes and the conserved sites bound by the seed region of miR-221/222, TargetScan Release 5.2 (www.targetscan.org) was used to predict the targets. To test the target genes as previously reported [30], a miRNA-target luciferase reporter assay was performed using a pEZX-MT01 target reporter plasmid containing the TIMP-2 3′ UTR to validate TIMP-2 as a direct target of miR-221/222. Moreover, we generated a mutant TIMP-2 3′ UTR (MUT-TIMP-2 3′UTR) reporter constructed by site-directed mutagenesis at the predictive target site of miR-221/222 in the wild-type TIMP-2 3′ UTR (WT-TIMP-2 3′UTR) as a control. HEK 293 cells were co-transfected in 24-well plates by Lipofectamine 2000 reagent with reporter plasmid (0.5 μg) and miR-221/222 mimic or control miRNA (50 nM). According to the manufacturer's instructions, the Dual-Luciferase Reporter Assay System (Promega, USA) was used to measure luciferase activity at 48 h after transfection. All the experiments were repeated in triplicate with duplicate samples.

### Statistical analysis

The data are expressed as the mean ± SD of at least three independent experiments. One-way ANOVA was used to determine significance. *P* < 0.05 was considered as statistically significant.

## Abbreviations

miRNA: microRNA
MMP-2: matrix metalloproteinase-2
MMP-9: matrix metalloproteinase-9
MMPs: matrix metalloproteinases
TIMP-2: tissue inhibitors of metalloproteinase-2
siRNA: small interference RNA
DMSO: dimethyl sulfoxide
OD: optical density
FCM: flow cytometry
UTR: untranslated region
ECM: extracellular matrix
